# Patient-Specific Plates for Genioplasty: A Case Report

**DOI:** 10.7759/cureus.38746

**Published:** 2023-05-08

**Authors:** Alladi Sneha, Murugesan Krishnan, Tharini Satheesh, Pradeep Dhasarathan, Hemavathy Muralidoss

**Affiliations:** 1 Oral and Maxillofacial Surgery, Saveetha Dental College and Hospitals, Chennai, IND

**Keywords:** genioplasty, virtual surgical planning (vsp), patient specific implant (psi), orthognathic surgery, digital planning

## Abstract

A 20-year-old male patient presented with a retruded chin and crowding of the upper front tooth region. The patient’s problem list included skeletal class II malocclusion, retruded chin, and shallow mentolabial sulcus. A treatment plan was curated using clinical examination, cephalometric analysis, and 3D measurements, which included the advancement genioplasty of 5 mm. Osteotomy cut was planned digitally by computer-aided surgical simulation technology (Dolphin Software, Dolphin Imaging Systems, California, USA) and then transferred to Geomagic Software (3D Systems, North Carolina, USA) where patient-specific plates were designed. The patient-specific plates were 3D printed using selective laser melting. Intraoperatively, the osteotomy cut was given using a surgical guide, and an advancement of 5 mm was performed, fixing the segments using patient-specific plates. The outcome was compared with the curated treatment plan to assess accuracy. The primary objective of the case report is to provide a digital method of the treatment plan and surgical accuracy in genioplasty using patient-specific plates.

## Introduction

The chin plays a significant role in facial harmony and balance, and any deformity of shape, size, spatial position, or proportion of the chin greatly affects the facial profile [1-3]. Genioplasty is a simple procedure that is widely practiced. The surgical results of genioplasty are influenced by where the osteotomy cut is placed and the direction in which the osteotomized segment is moved [4]. While attaining the golden proportion of the face, the gender and ethnic group of the patient should also be considered [5]. Traditionally for orthognathic surgery, conventional mock surgeries have been used for treatment planning based on the diagnosis made using clinical examination, radiograph, and cephalometric analysis. Hence, digital planning would provide accurate diagnosis and treatment planning. 

Various types of surgical templates have been used for genioplasty. These surgical guides or templates, which are fabricated using computer-aided surgical stimulation technology, often pose complications such as increased intraoperative time and bulkier templates interfering with surgical procedures. Additionally, the fabrication material of these templates is not rigid, and any deformation of the guide has a direct effect on the surgical outcome.

Patient-specific implants have been popularly advocated in various reconstructions, especially in craniomaxillofacial surgery. According to the literature, patient-specific implants/plates are validated for their accuracy in clinical and surgical outcomes [6,7]. Therefore, when computer-aided surgical simulation and patient-specific implants/plates are combined, they provide accurate clinical and surgical outcomes.

## Case presentation

**Figure 1:**
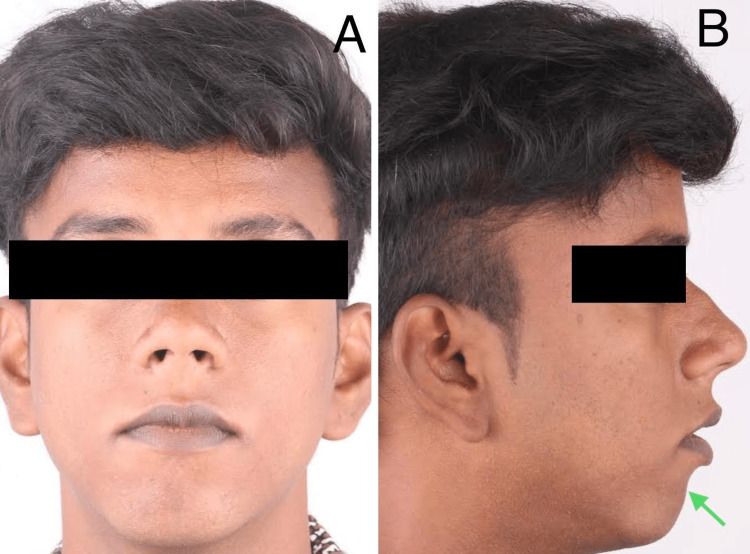
Pre-operative extraoral image. (A) Frontal; (B) profile depicting recessive chin with shallow mentolabial sulcus.

A 20-year-old male patient reported to the Department of Oral and Maxillofacial Surgery with a chief complaint of a retruded chin and crowding of the upper front tooth region. The patient presented with convex profile, incompetent lips, acute nasolabial angle, and retrognathic mandible (Figure 1).

**Figure 2:**
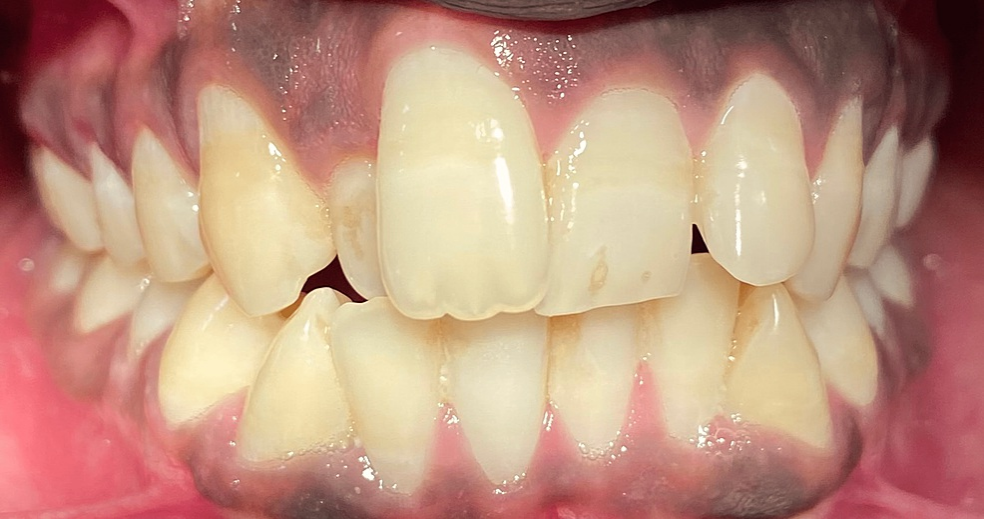
Pre-orthodontic occlusion. This image depicts occlusion before orthodontic treatment with a cross-bite in relation to 12.

**Figure 3:**
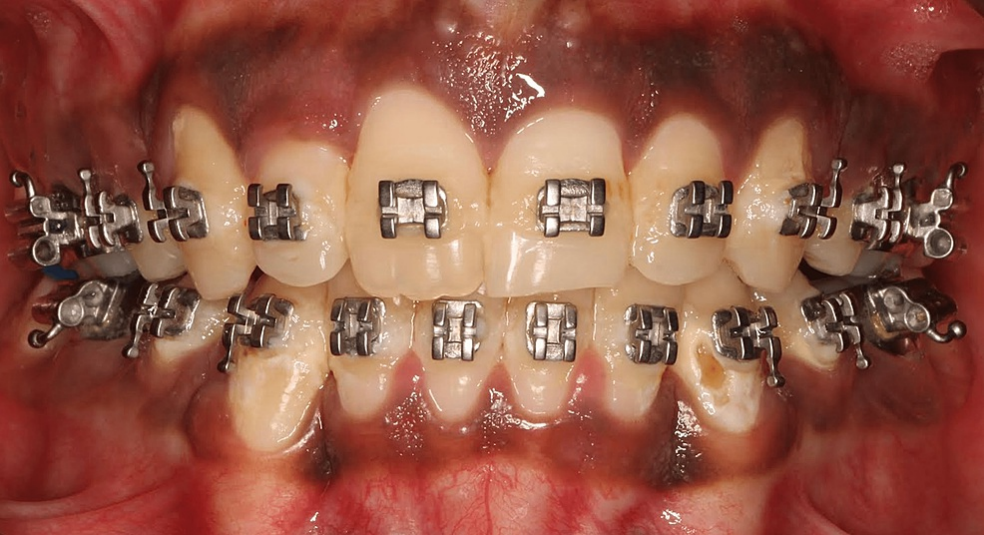
Post-orthodontic occlusion. This image depicts the orthodontically aligned occlusion prior to surgery.

On intra-oral examination, the molar relationship was class I occlusion with a cross-bite in relation to 12, a nasal breathing pattern, and abnormal circumoral muscle function. The problem list recorded was retrognathic mandible/skeletal class II malocclusion, retruded chin with shallow mentolabial sulcus, and a cross-bite in relation to 12 (Figures 2, 3). The patient was on orthodontic treatment for one year for the correction of malalignment of the dental segment. Though there is no role of pre-surgical orthodontics in genioplasty, pre-surgical orthodontics was done to address the patient's chief complaint, i.e., crowding of the upper anterior.

**Figure 4:**
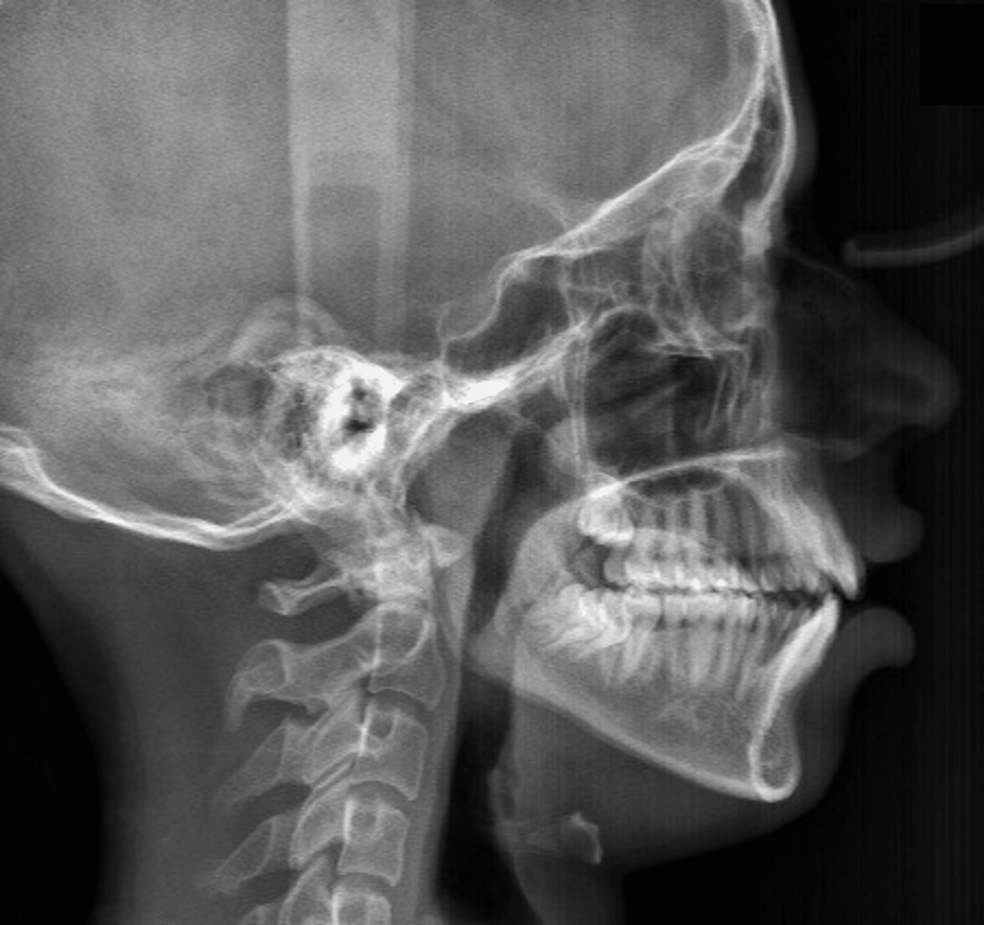
Pre-operative lateral cephalogram. This image depicts a lateral cephalogram prior to orthodontic treatment.

**Figure 5:**
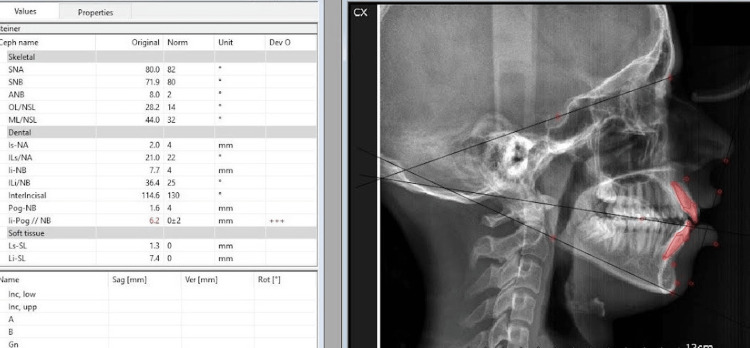
Pre-operative cephalometric analysis. This image depicts a retruded chin, according to Steiner's analysis.

**Figure 6:**
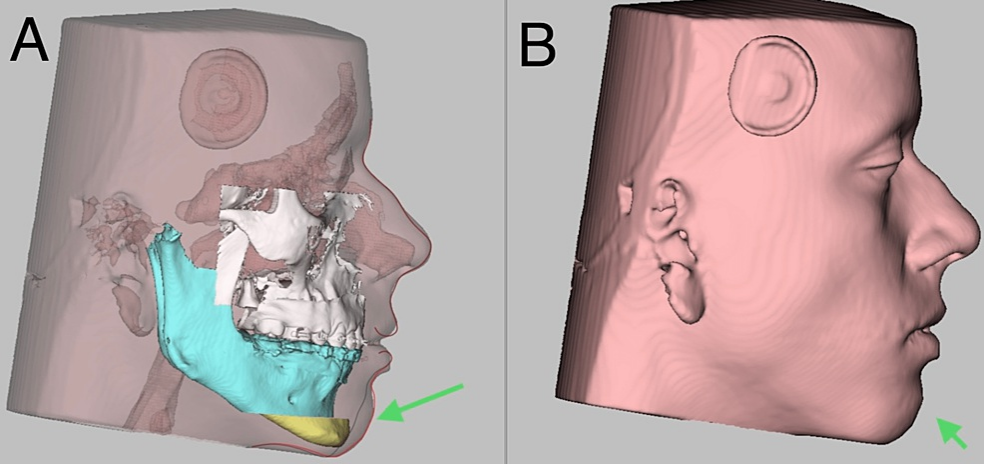
Virtual surgical planning. This is an image of virtual surgical planning that depicts: (A) postoperative hard tissue change; (B) postoperative soft tissue change.

A cone beam computed tomography of the full skull was taken, and intra-oral scanning was done to replicate the digital dental models (Figure 4). It was then merged with cone beam computed tomography to generate accurate dentate segments. Considering clinical examination, cephalometric analysis (Figure 5) and three-dimensional measurements, the osteotomy cut was virtually planned in Dolphin Software (Dolphin Imaging Systems, California, USA), extending from the right lower molar to the left lower molar and 6 mm below the mental foramen, where advancement genioplasty of 5 mm was performed (Figure 6) [8,9].

**Figure 7:**
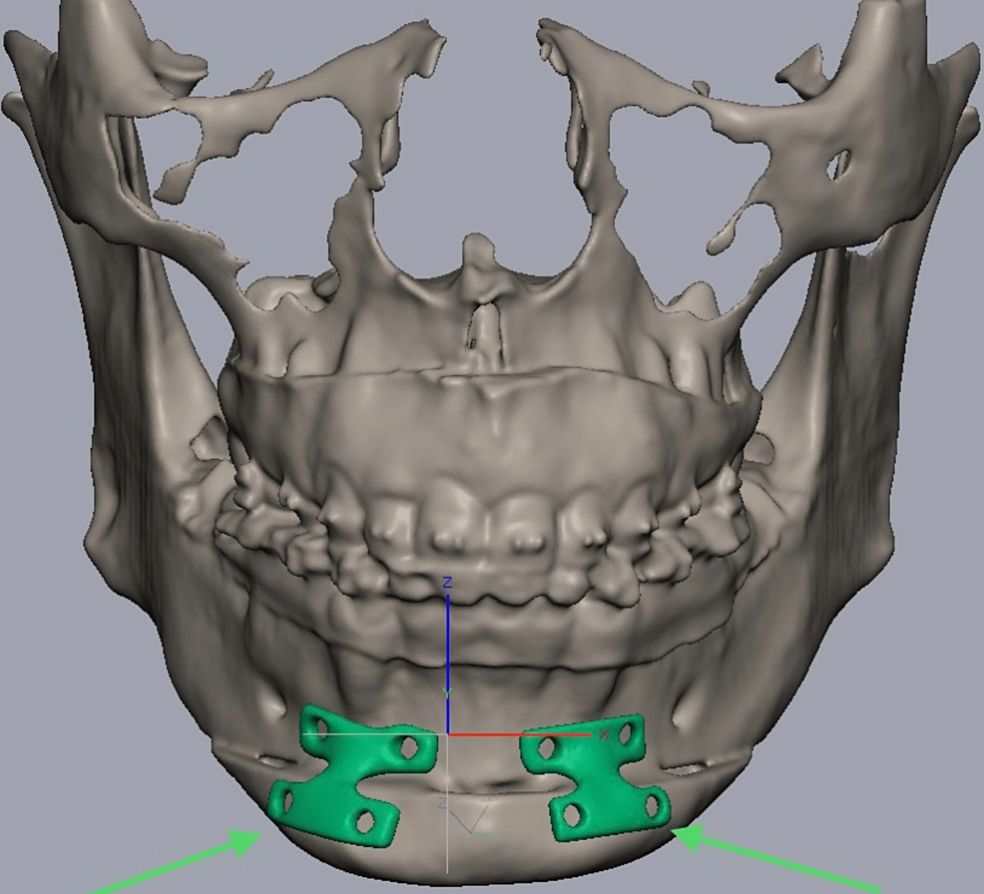
Patient-specific implant. This image shows a patient-specific implant planned using Geomagic Software.

The virtually curated surgical model was then transferred to Geomagic Software (3D Systems, North Carolina, USA) for the fabrication of the patient-specific plates. The stereolithographic model and the plates were then three-dimensionally printed using selective laser melting with a 2.5-mm thickness of titanium metal (Figure 7).

**Figure 8:**
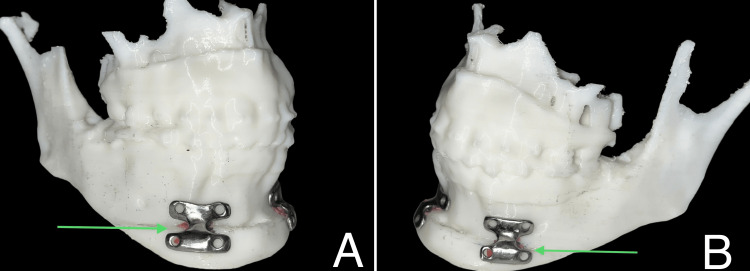
Stereolithographic model. This image depicts a stereolithographic model adapted with a 3-D printed patient-specific implant: (A) right; (B) left.

The plates were approved after perfect adaptation to the stereolithographic model. The patient-specific plates were then double-sterilized for the surgical procedure (Figure 8).

Surgical procedure

**Figure 9:**
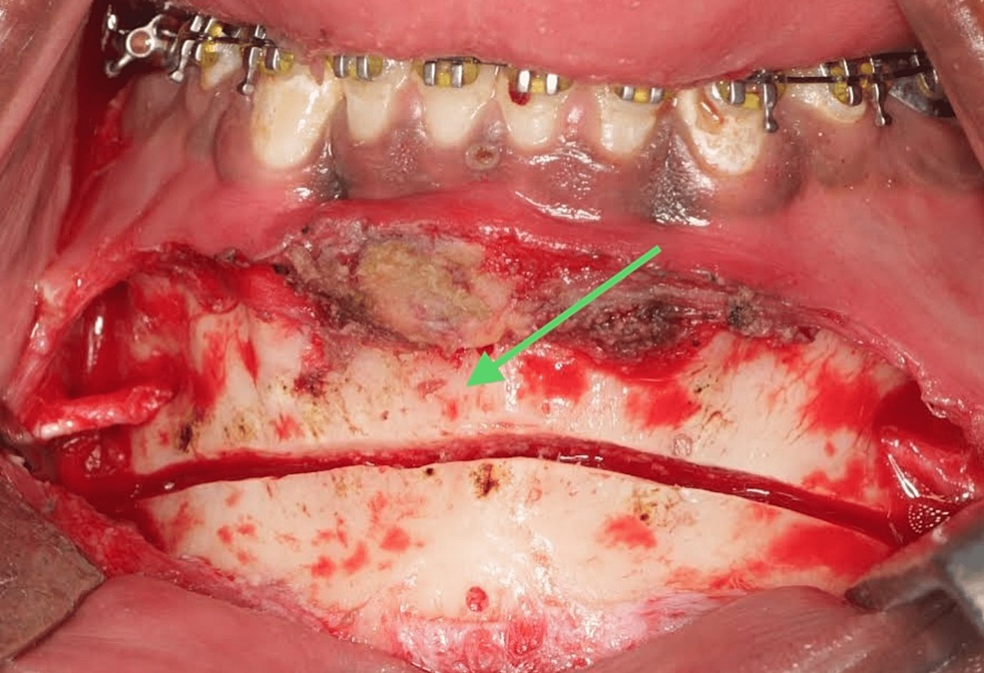
Osteotomy line. This image shows the osteotomy line marked using a surgical guide.

Under nasotracheal intubation with standard aseptic measures, a vestibular incision was made from 24 to 34 using a scalpel [10]. A full-thickness mucoperiosteal flap was elevated, and the mental nerve was identified and isolated bilaterally. A cutting guide was placed on the chin region. After achieving a perfect adaptation of the guide using a carbide taper fissure bur, the osteotomy line was placed (Figure 9).

**Figure 10:**
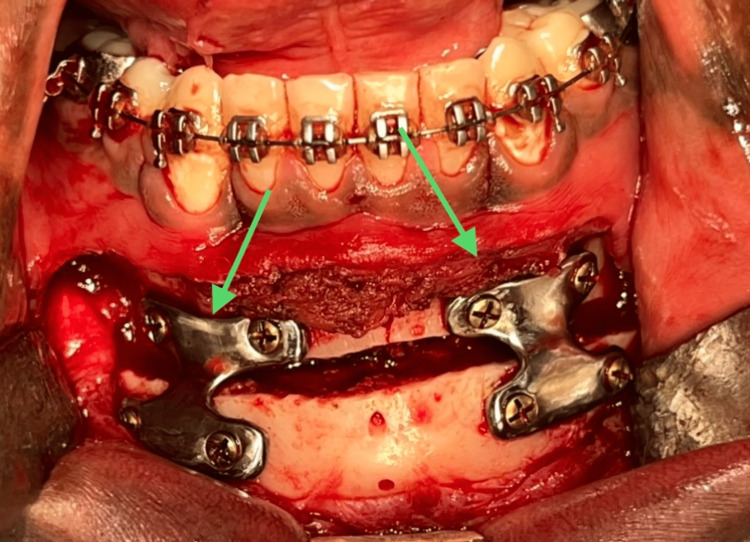
Fixation of patient-specific implant. This image depicts the fixation of a patient-specific implant using 2 mm titanium screws.

Following the removal of the cutting guide, osteotomy cuts were completed using a carbide taper fissure bur. The segment was then mobilized, and patient-specific titanium plates were placed in the digitally planned position of the osteotomized segment and fixed to the osteotomized segment using screws. The segment was then manipulated until the perfect adaptation of the plates was achieved and then fixed using screws while simultaneously manipulating the segment (Figure 10).

**Figure 11:**
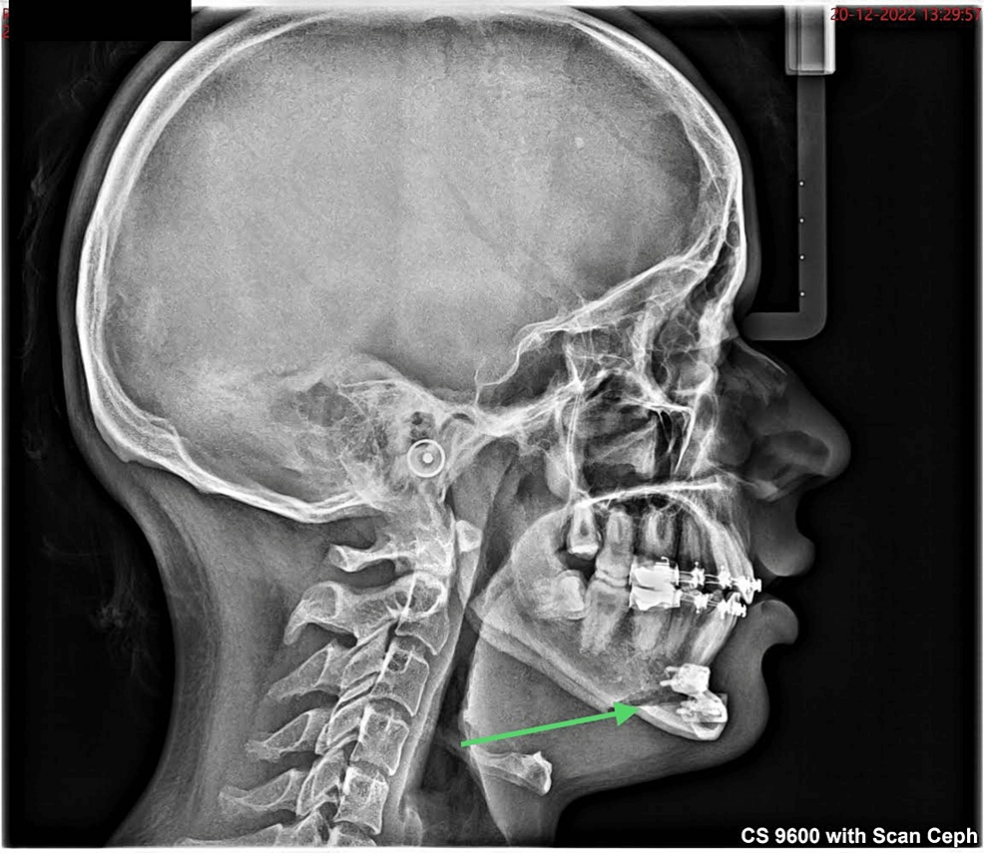
Post-operative lateral cephalogram.

Closure was done in layers using 3-0 polyglactin material. Postoperatively, a lateral cephalogram was taken to evaluate soft tissue and hard tissue changes (Figure 11). Postoperatively healing was uneventful.

**Figure 12:**
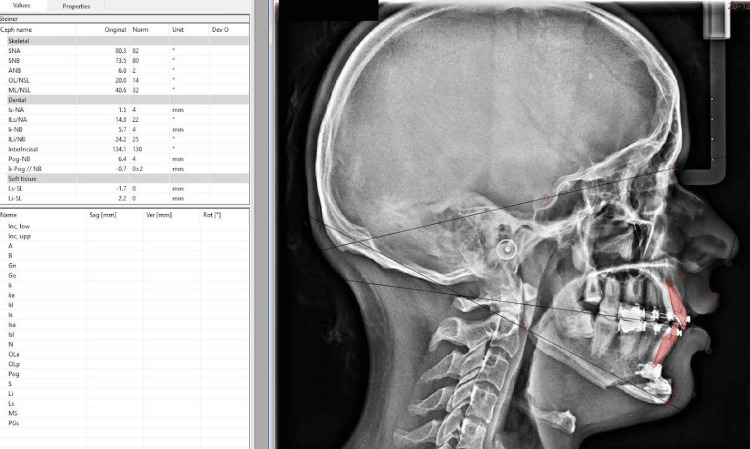
Post-operative cephalometric analysis. This image depicts Steiner's analysis following advancement genioplasty.

**Figure 13:**
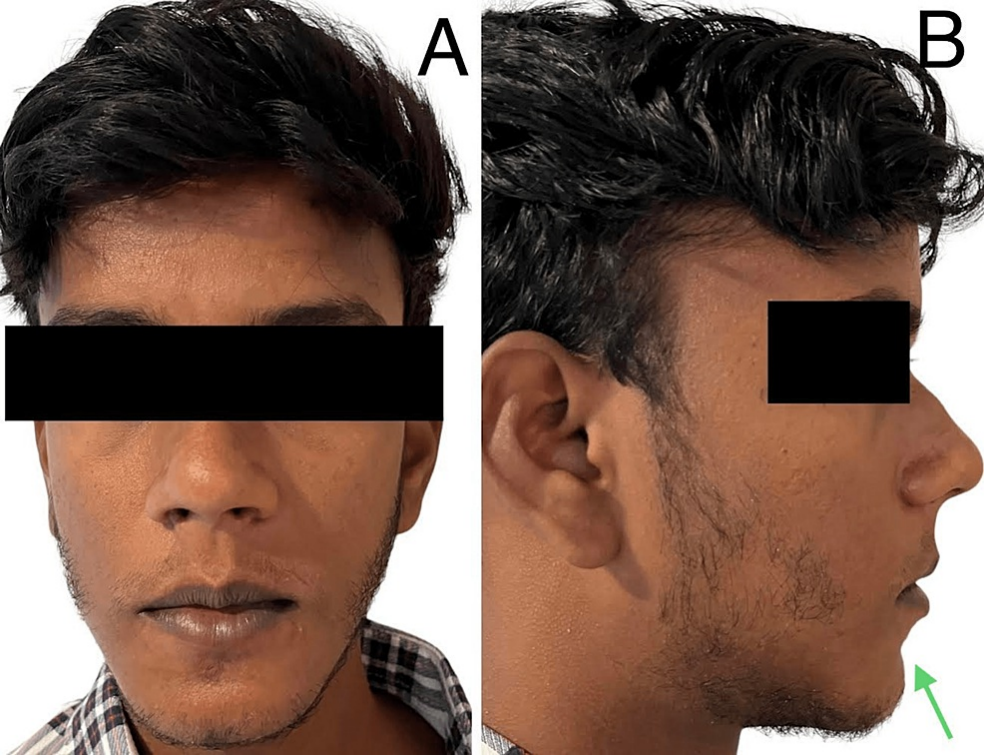
Post-operative extraoral image. This image shows a post-operative extraoral image: (A) frontal; (B) profile depicting the change in mentolabial sulcus.

Postoperatively, cephalometric analysis (Steiner's analysis) was done to compare and evaluate the accuracy of the surgical outcomes (Figures 12, 13).

## Discussion

When compared to traditional methods, patient-specific implants and plates eliminate the need for intraoperative measurements, the placement of holding screws for chin manipulation, or the bending of the implant intraoperatively. In patient-specific implants and plates, it can be designed according to the relationship between the inferior alveolar nerve and the roots of anterior teeth. Various complications have been reported in traditional methods, such as fixation failure and postoperative patient dissatisfaction [11,12]. The major advantage of computer-aided surgical simulation technology is that after digital mock surgery, the patient can visualize the postoperative changes, and there is a higher probability of patient satisfaction post-operation. In studies where the usage of computer-aided design or computer-aided manufacture templates for genioplasty has been reported [13-15], these templates posed a major drawback where the tooth-borne templates work as a locking unit and are hence bulkier, interfering with the surgical procedure. In patient-specific plates, the plate is placed in its unique position, so there is no need for a locking unit to stabilize the plate. Another drawback includes the use of a repositioning guide, which increases intraoperative time.

The disadvantage of patient-specific implants and plates is their time and cost-effectiveness. Preoperatively, digital planning and fabrication are time-consuming compared to the fixation of prefabricated miniplates. Another disadvantage that is applicable to all patient-specific implants and plates is that changes in the treatment plan are not possible once the implant or plate is fabricated, as these plates can barely be bent and reshaped. The biomedical properties of titanium have already been proven in the past decades, which stand for their biocompatibility, resistance to corrosion, and body fluids. The biomechanical aspects of these patient-specific implants share similar properties to those of standard titanium miniplates, such as stress distribution and stress shielding effects.

Various studies focus on clinical and surgical outcomes, but a common complication encountered is the gaping of sutures and infection secondary to implant fixation. Recent advancements of suture material reportedly provide better wound healing, but due to insufficient literature on the same, the closure was done using polyglactin suture [16-18]. Further studies are required to assess the infection secondary to implant or plate fixation, predominantly in patient-specific implants or plates.

## Conclusions

To conclude, patient-specific plates provide clinical and surgical accuracy in spite of its preoperative planning. Using patient-specific plates makes surgical procedures simplified by eliminating intraoperative measurements and manipulation. Additionally, there is a higher probability of patient satisfaction post-operation. Considering their pros and cons, patient-specific implants/plates using computer-aided surgical simulation technology and computer-aided design and manufacture technology have a promising potential to gain popularity and acceptance in the upcoming years.
